# A Molecular Troika of Angiogenesis, Coagulopathy and Endothelial Dysfunction in the Pathology of Avascular Necrosis of Femoral Head: A Comprehensive Review

**DOI:** 10.3390/cells12182278

**Published:** 2023-09-14

**Authors:** Monica Singh, Baani Singh, Kirti Sharma, Nitin Kumar, Sarabjit Mastana, Puneetpal Singh

**Affiliations:** 1 Division of Molecular Genetics, Department of Human Genetics, Punjabi University, Patiala 147002, India; singhmonica2017@gmail.com (M.S.);; 2 Human Genomics Laboratory, School of Sport, Exercise and Health Sciences, Loughborough University, Loughborough LE11 3TU, UK

**Keywords:** avascular necrosis of femoral head, impaired angiogenesis, coagulopathy, endothelial dysfunction, bone disease, skeletal abnormality

## Abstract

Avascular necrosis of the femoral head (ANFH) is a painful disorder characterized by the cessation of blood supply to the femoral head, leading to its death and subsequent joint collapse. Influenced by several risk factors, including corticosteroid use, excessive alcohol intake, hypercholesterolemia, smoking and some inflammatory disorders, along with cancer, its clinical consequences are thrombus formation due to underlying inflammation and endothelial dysfunction, which collaborates with coagulopathy and impaired angiogenesis. Nonetheless, angiogenesis resolves the obstructed free flow of the blood by providing alternative routes. Clinical manifestations of early stage of ANFH mimic cysts or lesions in subchondral bone, vasculitis and transient osteoporosis of the hip, rendering it difficult to diagnose, complex to understand and complicated to cure. To date, the treatment methods for ANFH are controversial as no foolproof curative strategy is available, and these depend upon different severity levels of the ANFH. From an in-depth understanding of the pathological determinants of ANFH, it is clear that impaired angiogenesis, coagulopathy and endothelial dysfunction contribute significantly. The present review has set two aims, firstly to examine the role and relevance of this molecular triad (impaired angiogenesis, coagulopathy and endothelial dysfunction) in ANFH pathology and secondly to propose some putative therapeutic strategies, delineating the fact that, for the better management of ANFH, a combined strategy to curtail this molecular triangle must be composed rather than focusing on individual contributions.

## 1. Introduction

**Figure 1:**
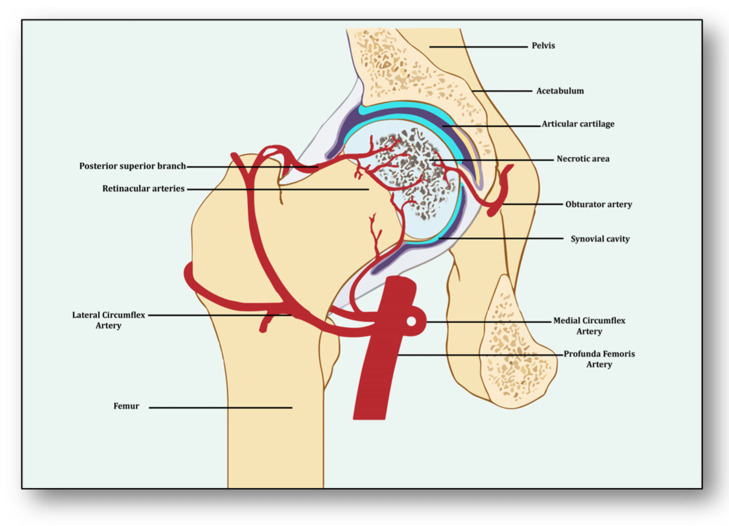
Impaired blood supply causing necrosis of the femoral head.

Avascular necrosis of the femoral head (ANFH) is a debilitating condition identified as the death of the bone tissue due to compromised blood supply to the subchondral bone and its delayed regeneration [1]. Being a multifactorial and complex disease, its pathogenesis remains unclear. Several risk factors, including excessive alcohol intake; corticosteroid use; some medical conditions such as lupus, sickle cell disease and clotting disorders; and hormonal imbalances along with cancers and medical procedures like high-dose radiation therapy, hip surgery, bone marrow transplant and genetic factors participate in its pathology [2]. The effect of these risk factors generally induces dysfunction in the molecular pathways which initiate, develop and progress ANFH [3]. Three important pathways which may play a crucial role in ANFH pathology are impaired angiogenesis, coagulopathy and endothelial dysfunction [4]. Understanding the intricate and unforeseen interplay among these three molecular mechanisms is essential for unraveling the underlying pathways, which will help devise effective therapeutic strategies for ANFH [4]. Largely, ANFH is caused by hindered or cessation of blood supply to the femoral head, leading to necrosis because of an inadequate supply of oxygen and nutrients to the bone tissue (Figure 1). In response, a cascade of events is initiated for restoring impaired blood flow.

Consequently, angiogenesis, the process of new blood vessel formation, is triggered as a compensatory response to the compromised blood supply [5]. It behaves as a double-edged sword. On one side, it tries to establish alternative routes for the free flow of blood to the necrotic area of the femoral head by forming new vessels and a capillary network, which may be structurally abnormal and leaky, causing edema, hence failing to adequately restore blood flow. On the other side, if angiogenesis is excessive and uncontrollable, then it may cause unusual bone remodeling, thereby exacerbating the problem of ANFH [6].

Coagulopathy is the condition of disturbed homeostasis of procoagulant and anticoagulant factors that promotes excessive clot formation and demotes thrombolysis [7]. It exaggerates ischemia and internal bleeding because of reduced production of fibrinolytic enzymes such as tissue plasminogen activator (tPA) [8]. Furthermore, coagulopathy participates in the formation of intravascular and microvascular thrombi, thereby obstructing the blood flow, exacerbating the tissue ischemia and delaying the tissue repair [9].

Endothelial dysfunction represents the impairment of the normal functioning of endothelial cells, thereby disrupting the delicate balance of vasoconstriction and vasodilation [10]. This dysfunctional state distorts the blood flow regulation and the release of vasoactive molecules, which fails to resolve the exigency of the blood supply to the femoral head, leading to ischemia, subsequent bone tissue damage and worsening the condition by initiating a pro-inflammatory state [11]. Several inflammatory cytokines and adhesion molecules promote the recruitment of immune cells to the affected area, thereby perpetuating the release of destructive enzymes and reactive oxygen species, causing further damage to the bone tissue [12]. Additionally, endothelial dysfunction promotes a prothrombotic state within compromised blood vessels and delays the fibrinolysis of the clots, depriving the affected area of vital nutrients and oxygen for regeneration and repair [13].

The present review aims to investigate these perplexing molecular mechanisms and signaling pathways which participate individually as well as collaboratively in the initiation, progression and worsening of ANFH pathology [14]. Knowledge obtained from this review will help in paving the way for innovative interventions and offers a comprehensive but lucid list of therapeutic strategies for its early identification, leading to better management of this disease with its severe ramifications.

## 2. Angiogenesis: Sprouting, Splitting and Stabilization

Angiogenesis is a process of developing new vessels from pre-existing vessels, and these vessels are considered to be the foremost organ in embryo development [15]. Vessels may develop, remodel and grow in different ways. They may sprout from already present vessels (sprouting angiogenesis) or develop by splitting from previously prevailing arteries or capillaries upon receiving the angiogenic stimuli (intussusceptive) [16]. They may enlarge and elongate from the coalescence of capillaries (coalescent angiogenesis), promoting the rapid expansion of the vasculature. Vessels may remodel themselves to increase their luminal diameter when more blood flow is required or there is a need to develop collateral bridges to provide alternative routes for the free flow of blood (arteriogenesis) [17]. Unlike angiogenesis, new blood vessels may be formed from the blood islands where no pre-existing vessels are present (vasculogenesis) [16]. All newly formed vessels must be mature and stable, otherwise they may be abnormal and leaking, further causing problems of local hematoma and edema [18]. These become stable due to the signaling molecules arranging pericytes overlaid on endothelial cells and the formation of the basement membrane, whereby junctions are established to allow optimum blood flow [19]. Angiogenesis has both beneficial and detrimental effects on health and disease, which makes it a potent hotspot for both pro-angiogenic and anti-angiogenic drug targets [20].

From the perspective of avascular necrosis, regulated angiogenesis is beneficial for collateral circulation to the necrotic area and its repair, whereas dysregulated angiogenesis is harmful [21]. The femur is a specially structured and highly vascularized bone, the longest in the human body. Its mechanical strength, recuperation, repair, regeneration and remodeling depend upon vascular health, which helps in supplying incessant blood and providing adequate oxygen, nutrients, growth factors and osteoprogenitor cells to the bone [22]. Consequently, angiogenesis is expected to revascularize, reperfuse and resorb the necrotic area. Branching from the circulatory system, the nutrient artery is the largest blood vessel that enters the medullary cavity and supplies almost half of the total blood volume to the femur [23]. At the proximal end, it forms anastomoses with perforating arteries, whereas at the distal side it merges with the profunda femoris artery. It extends longitudinally to the bone and divides into the lateral femoral circumflex artery and medial femoral circumflex artery [24]. Both branches of lateral femoral circumflex arteries feed the femur head region via lateral epiphyseal arteries and the neck region through posterior superior retinacular arteries [25]. The ligament of the femur head is also supplied by the anterior branch of the obturator artery of the hip bone, which traverses through the inferior part of the pubic ramus and anastomoses with the femoral artery and medial femoral circumflex artery [26] (Figure 1).

With the advent of three-dimensional high-resolution imaging, a new aspect of the anatomy of vessels linking bone vasculature and bone marrow has been discovered [27]. This newly discovered vascular system comprises arterioles, venioles and capillaries, which collectively have been named as transcortical vessels (TCVs), which have shed light on the connection between endosteal and periosteal circulation [28]. A new subtype of blood vessels expressed from endomucin (Emcn) and a cluster of differentiation 31 (CD31) on endothelial cells in the bone known as type H has been identified lately [29]. Present in endosteum and metaphysis, this vessel is considered to be full of mesenchymal and osteoprogenitor cells, which mediate subchondral remodeling by coupling angiogenesis and osteogenesis [30]. Whether it contributes to ANFH pathology remains to be clarified as crosstalk between subchondral bone and articular cartilage during ischemia is unclear [31]. Obstruction or ischemia may take place in any of these arteries, but lateral and medial branches of the femoral circumflex or retinacular arteries are largely involved [32].

## 3. Angiogenesis: A Predominant Pacifier in Avascular Necrosis

Several stimuli are received by endothelial cells from the local environment, prompting them to initiate angiogenesis [33]. These signaling stimuli augment endothelial cell activation and their migration along with apoptotic resistance, cytoskeletal reorganization and endothelial cell proliferation. Firstly, angiogenesis is triggered in response to ischemia, which is a dynamic process leveraging the equilibrium between pro-angiogenic and anti-angiogenic factors, resulting in the expansion of the vascular network [34]. Several vasculature and bone-derived angiogenic factors and stimulators play a role in angiogenesis to counterbalance the exigencies of nutrients and oxygen to the necrotic area [35]. They are hypoxia inducible factor-1α (HIF1-α), vascular endothelial growth factor (VEGF), vascular endothelial growth factor receptor (VEGFR), neuropilin 1 (NRP1), angiopoietin 1 (ANGPT1), angiopoietin 2 (ANGPT2), platelet-derived growth factor (PDGF), transforming growth factor-β (TGF-β), C-C motif chemokine ligand-2 (CCL-2), integrins αvβ3, αvβ5., vascular endothelial cadherin (VE-cadherin), cluster of differentiation 31 (CD31), plasminogen activators, inhibitor of DNA binding-1/inhibitor of DNA binding-3 (ID1/ID3), bone morphogenetic protein (BMP), prostaglandins (PTG), adenosine, pleiotrophin (PTN), delta-like canonical Notch ligand 4-Notch-Noggin (DLL4-NOTCH-NOG), receptor activator of nuclear factor-kappa β ligand/receptor activator of nuclear factor-kappa β/osteoprotegerin (RANKL/RANK/OPG), semaphorin (SEMA), nitric oxide (NO) and matrix metalloproteinases (MMPs) [36]. Nonetheless, molecular pathways between vasculature and bone interact and collaborate to initiate angiogenesis–osteogenesis coupling, which is required for the overall regeneration and repair of the necrotic area [37]. Endothelial cells along with pericytes initiate endocrine signaling, whereas osteoblasts and osteoclasts trigger angiogenesis to manage and maintain vasculature.

After ischemia, hypoxic conditions emerge, which induce HIF-1α and VEGF in response [38]. It has been considered that HIF-1α is the precursor for the upregulation of VEGF, which is corroborated by the finding that transplantation of HIF1-α with transgenic bone marrow cells onto the necrotic area upregulated VEGF and increased angiogenesis, resulting in the repair of the necrotic area [35]. The nuclear signal transduction augments the translocation of HIF1-α to form a complex with HIF1-β and transcriptional co-activator E1A-associated protein/CREB binding protein (p300/CBP), which helps them to bind with the hypoxia response element [39]. It translates into the activation of several angiogenic genes such as VEGF, ANGPT-2 and nitric oxide synthase (NOS). VEGF plays the main role in bone remodeling via differentiation of osteoblasts and promoting endothelial cells at the affected area [40]. Besides the involvement of other forms of VEGF (VEGF-B, VEGF-C, VEGF-D and placenta growth factor), VEGF-A is primarily involved in angiogenesis and vasculogenesis during ischemic insult by binding and activating both VEGF receptors, i.e., VEGFR-1 and VEGFR-2, for vascular permeability, cell migration, vascular function and vessel maintenance [41].

**Figure 2:**
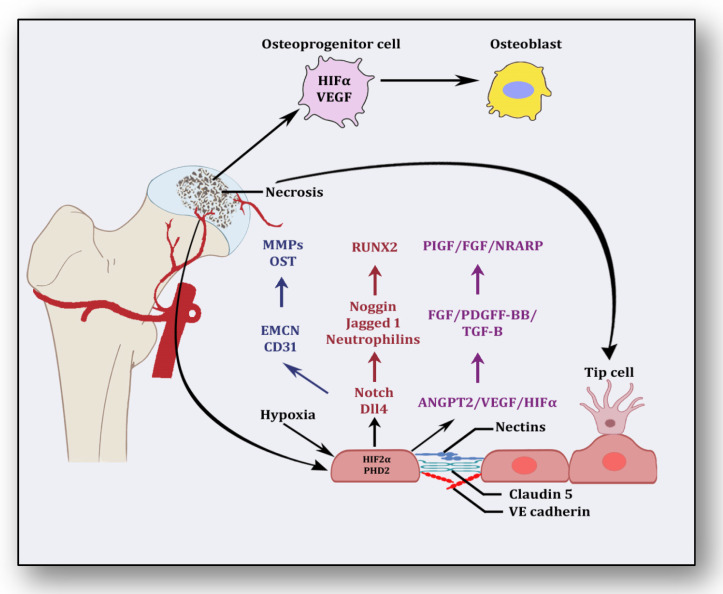
Angiogenic stimuli at cartilage and endothelium.

Besides promoting endothelial cell differentiation, migration and proliferation, VEGF initiates the recruitment of bone-marrow-derived endothelial progenitor cells at the affected area [42] (Figure 2). Consequently, it promotes morphogenesis of the growth plate, blood vessel formation and remodeling of the affected cartilage [43]. The mechanism of VEGF-induced angiogenesis is essential in cartilage revascularization at both early stage and end stage after necrosis [44]. It is expressed in the edematous area of the necrotic zone and plays a significant role in the repair of the ongoing hypoxia-induced osteonecrotic area [45]. In the absence of VEGF, angiogenesis has been observed to be arrested, and the process of trabecular and cortical bone repair is significantly attenuated [46]. Moreover, it directly influences the osteoblast activity by increasing nodule formation and alkaline phosphatase, thereby promoting mineralization in a dose-dependent manner [47]. This suggests that the upregulation of VEGF in osteoblasts during hypoxia participates in and contributes to the healing process by promoting initial calcification at the site of injury [48].

Endothelial cells have oxygen sensors and hypoxia-inducible factors: hypoxia-inducible factor-2 alpha (HIF-2α) and prolyl hydroxylase domain 2 (PHD2) [49]. After obtaining subtle hypoxemic stimuli, vessels start readjusting their size by vasodilation to receive blood flow. These endothelial cells adjust as a monolayer of phalanx cells and establish interconnections through the adhesion activity of VE-cadherin and claudins [50]. These layers are overlaid with pericytes, which signal for survival to VEGF and ANGPT-1. Angiogenic signaling is initiated due to hypoxia, and pericytes are separated from the vessel wall and detach from the basement membrane via proteolytic degradation controlled by MMPs [51]. Consequently, interconnections are loosened, and the naked vessel starts enlarging. Extracellular matrix support is provided by VEGF signaling, whereby endothelial cells migrate owing to integrin signaling [52]. Angiogenic molecules such as VEGF and FGF are released due to the action of proteases. Special endothelial cells, tip cells in the presence of factors such as VEGF receptors, DLL4-NOTCH, Jagged1 and neuropilins, form the tube-like structure and inhibit endothelial cells from migrating toward angiogenic signals [51]. Prompted and mediated by the signaling of NOTCH, WNTs, Placental growth factor (PlGF), FGF, NOTCH-regulated ankyrin repeat protein (NRARP), VE-cadherin, VEGF, Hedgehog and CD34, the tip cells of the flanking region are established as stalk cells, which split and extend to form lumen [53]. Local environmental stimuli are sensed by filopodia of the tip cells with the help of ephrins and semaphorins, whereas angiogenic signals are sensed by HIF-1α. In response, myeloid cells establish a link with another vessel, allowing the free flow of the blood [51]. These vessels must be stable and properly formed, otherwise they become leaky and promote hypoxia and ischemia [18]. To acquire maturity and stability, endothelial cells become quiescent, and these signals are responded to by NOTCH, PDGFB, ANGPT-1, TGF-β and ephrin-B2 to form a layer of pericytes on endothelial cells [54]. Some protease inhibitors such as tissue inhibitor metalloproteinases (TIMPs) and plasminogen activator inhibitor-1 (PAI-1) arrange the basement membrane, whereby junctions are formed to provide ideal blood flow. Vessels may regress if perfusion is not established [55].

Perturbed homeostasis due to an imbalance between pro-angiogenic and anti-angiogenic factors leads to abnormal angiogenesis, which may enhance the problem of necrosis due to its proinflammatory and profibrotic signaling and inability to resolve vascularization [56]. Overtly dilated or constricted conduits due to abnormal branching angles have been observed when anti-angiogenic factors such as endostatin and angiostatin are abundantly produced in the case of systemic sclerosis [57]. Interestingly, VEGF may have two isoforms, i.e., VEGF165a and VEGF165b, due to alternative splicing in the pre-mRNA terminal exon [58], a probable reason that some studies have observed impaired angiogenesis even in the presence of higher levels of VEGF [59]. When angiogenesis is coupled with proinflammatory and profibrotic signaling, a pro-angiogenic isoform of VEGF (VEGF165) can be switched to an anti-angiogenic isoform (VEGF165b) in platelets [60]. Causes and consequences of abnormal or impaired angiogenesis for the risk of necrosis of the femoral head during and post-ischemia have not been investigated so far; nonetheless, concerns are similar, and hence clarifications are convincing, that angiogenesis resolves while impaired angiogenesis worsens the clinical outcome of ANFH.

## 4. Coagulopathy: A Culprit Alliance of Thrombophilia and Hypofibrinolysis

Following the revelation from the first study by Hamilton et al. in 1965, several studies have endorsed that the pathology of osteonecrosis resulting from vascular ischemia is strongly influenced by coagulopathy [61]. Intravascular coagulation and thrombosis coupled with excessive thrombophilia and hypofibrinolysis are the major reasons [62]. Thrombophilia, sometimes called hypercoagulability, is an abnormality of the clotting mechanism which promotes thrombus formation within walls of blood circulatory vessels. Thrombophilia predominantly develops into deep venous thrombosis (DVT) and pulmonary embolism (PE), two chief reasons for cardiovascular morbidity and mortality. Both of these hypercoagulable conditions are termed venous thromboembolism (VTE). VTE deteriorates fibrinolytic machinery causing hypofibrinolysis, an abnormal condition whereby clot-resolving factors are dysregulated and clot-forming conditions are promoted. Fibrinolysis is the process of breaking down thrombus or clots and is strictly regulated by activators such as tissue plasminogen activator (tPA) and urokinase-type plasminogen activator (uPA) as well as inhibitors like tissue factor plasminogen inhibitor (TFPI) and plasminogen activator inhibitor-1 (PAI-1) and a fibrinolytic protease, plasmin. Plasminogen converts to plasmin via FXIa, FXIIa and kallikrein. This step triggers fibrinolysis by activating tPA within endothelial cells and uPA through the urinary epithelium, monocytes and macrophages. These factors play a significant role in breaking down and clearing clots from vasculature, whereas hypofibrinolysis (decreased levels of tPA and increased levels of PAI-1) impairs clot breakdown and prolongs its clearance. Several primary factors such as low levels of activated protein C (APC), protein S, factor V Leiden, activated protein C resistance (APCR), low levels of tPA or high levels of PAI-1, high levels of von Willebrand factor (vWF), high levels of lipoprotein(a) (Lp(a)) and homocystinuria along with secondary factors such as antiphospholipid antibodies, corticosteroid use, systemic lupus erythematosus (SLE) and caisson disease hemoglobinopathies, hemato-oncological diseases such as chronic myelogenous leukemia, acute lymphoblastic leukemia and multiple myeloma also participate in and contribute to causing hypofibrinolysis [62,63,64,65,66,67,68,69,70].

**Figure 3:**
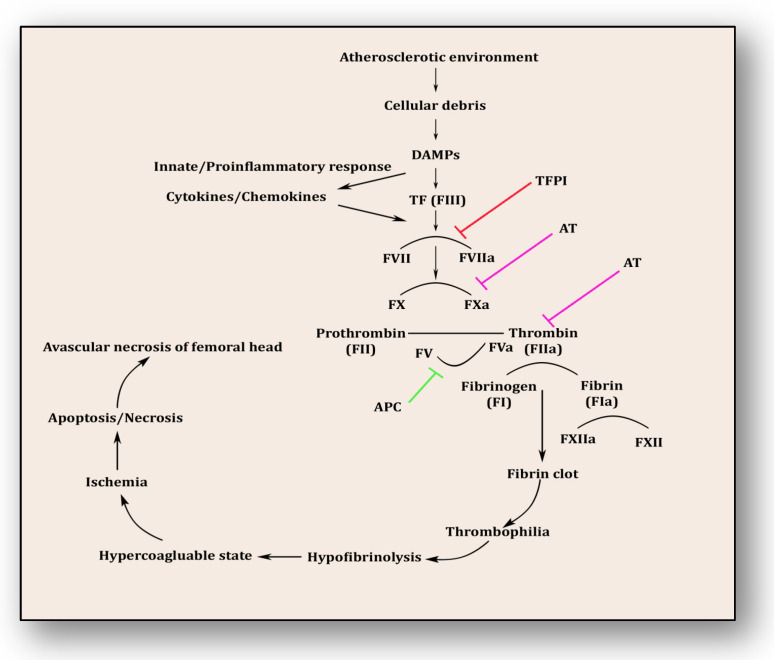
Coagulation pathway in the pathology of avascular necrosis of the femoral head.

The coagulopathy cascade comprises a localized and speedy activation of inactive serine proteases (clotting factors) sequentially to generate thrombin resulting in clot formation (fibrin mesh) [71]. This pathway is triggered by sub-endothelial mural cells and fibroblasts of vascular adventitia. Coagulation may also trigger due to low levels of circulating polymorphonuclear neutrophils and monocytes/macrophages [72]. The first and foremost trigger is the exposure of tissue factor (TF) due to the severity of endothelial cell damage [73]. This exposed TF combines with factor VII to activate it to FVIIa, culminating in a sequence of activating factors such as FIX to FIXa and FX to FXa [7]. FXa turns prothrombin to thrombin, which further activates FV and FVIII to FVa and FVIIIa, a step responsible for converting prothrombin to thrombin by activating FX to FXa [7]. Furthermore, this thrombin-mediated fibrin clot is solidified by FXIa and interlinked by FXIIIa [74]. Activated platelets aggregate to form this clot as TF-presenting cells, ultimately augmenting coagulation and thrombus formation [75]. This process is simultaneously regulated by inhibitors of coagulation so that clot formation is not unnecessary and remains localized. TFPI, anti-thrombin (AT) and protein C are three major inhibitory molecules that check and resolve excessive coagulation within vessel walls [76] (Figure 3). Furthermore, the fibrinolytic pathway mediates vessel wall agility, integrity and healing.

Innate immune response and inflammatory reaction play roles simultaneously within endothelium to resolve ensuing damage and support the healing process. Phagocytes, antigen-presenting cells, monocytes and neutrophils are prompted upon encountering danger-associated molecular patterns (DAMPs) and pathogen-associated molecular patterns (PAMPs) [77]. Consequently, activated monocytes and neutrophils initiate an immune response against cellular debris referred to as immunothrombosis, which emerges in response to atherosclerotic connotations within vessel walls. Vascular debris due to damaged endothelium and plaque formation is sensed by PAMPs and DAMPs and in response initiates tissue factor expression on monocytes and neutrophils, furthering immunothrombosis [78]. This unresolved and uncontrolled immunothrombosis forms disseminated intravascular coagulation (DIC). Side by side, DAMPs and PAMPs induce a proinflammatory cascade along with antimicrobial cytokines and chemokines by upregulation of intracellular cell adhesion molecules (ICAMs) and vascular cell adhesion molecules (VCAMs) [77,78]. This mechanism plays a significant role in resolving the ischemic insult and repairing tissue damage. DAMPs were efficiently disposed of by initiating a complement activation cascade triggered by membrane-anchored proteins and soluble regulators.

Etiopathology of osteonecrosis of the femoral head is influenced by two molecular pathways, i.e., thrombophilia and hypofibrinolysis. Their clinical causative consequences are heightened intraosseous venous pressure and reduced arterial flow supporting hypoxia-induced ischemic insult in the bone vasculature. Both familial and acquired thrombophilia/hypofibrinolysis contribute to osteonecrosis of the jaw/hip in both children and adults [79]. Primarily, glucocorticoids attenuate fibrinolytic activity by increasing PAI-1 levels and decreasing tPA levels [80]. PAI-1 and tPA work in unison to resolve thrombosis by increasing fibrinolysis because their PAI-1/tPA complex inhibits plasmin generation from plasminogen. Glucocorticoids increase the functional activity of PAI-1 and reduce tPA levels, thereby triggering a hypercoagulable state [81,82]. Resulting in osteonecrosis of the femoral head, this thrombophilia-hypofibrinolysis duo is further supported by higher levels of fibrinogen and Lp(a) promoting platelet activation resulting in delayed lysis of thrombosis [83]. Furthermore, P1A1/A2 polymorphism in glycoprotein IIIa, lupus anticoagulant, reduced levels of protein C, S and anti-thrombin III along with cardiolipin antibodies contribute to the thrombophilia-hypofibrinolysis axis-induced osteonecrosis of the femoral head. In secondary ANFH, endothelial dysfunction rather than thrombophilia collaborates with hypofibrinolysis in worsening ANFH outcomes [84].

## 5. Endothelial Dysfunction: Holding Hands with Inflammation

**Figure 4:**
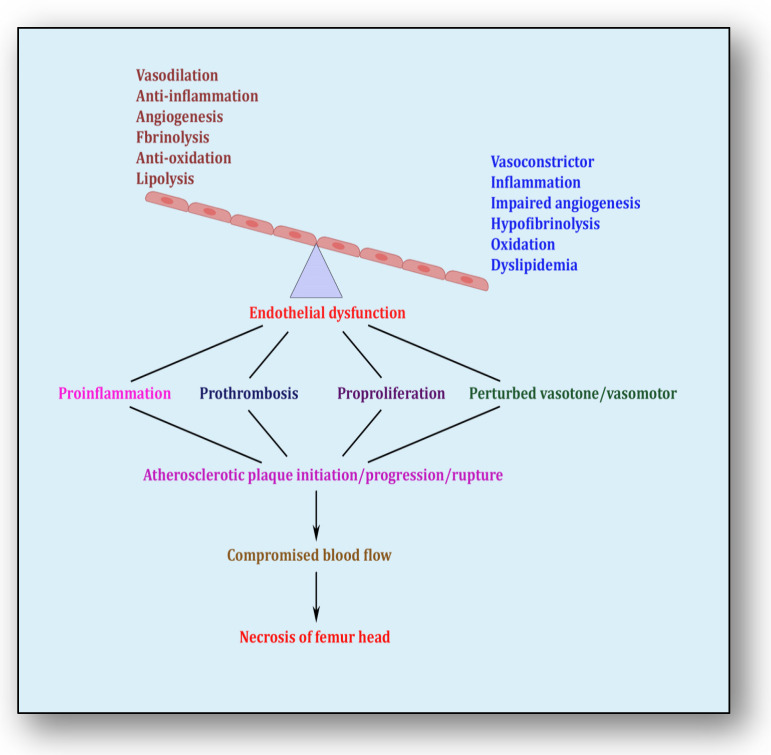
Endothelial dysfunction showing impaired homeostasis and risk pathways.

The endothelium is a cell lining positioned on the inner surface of the blood vessels dividing circulating blood from the tissue. In response to various physical and chemical stimuli, such as perturbed blood flow, excessive intramural pressure, oxidative stress, cellular damage, high levels of homocysteine, hyperlipidemia, toxic chemicals and bacterial/viral infections, it initiates endocrine, paracrine and autocrine functions to produce vasodilators such as nitric oxide (NO), prostacyclin (PGI2) and endothelium-derived hyperpolarizing factors (EDHFs) and vasoconstrictors such as endothelin-1 (ET-1) and thromboxane-A2 (TXA2) [85]. The endothelium regulates homeostasis by maintaining the balance between vasodilators and vasoconstrictors, anticoagulants and procoagulants, inflammatory and anti-inflammatory molecules, oxidants and antioxidants as well as profibrinolytics and antifibrinolytics (Figure 4) [86]. Due to several risk factors, this homeostasis is lost and is termed endothelial dysfunction. In prolonged endothelial dysfunction, cholesterol microcrystals, monocytes and lymphocytes enter layers of endothelium and initiate inflammatory response, which helps in the formation of fatty streaks resulting in plaque setup, its progression and rupture. Plaque rupture expounds thrombus formation, which couples with coagulation cascade, resulting in atherogenesis and vascular ischemia [87]. Alluding to its contribution to the pathology of several diseases, endothelial dysfunction has been recognized as the diagnostic and prognostic marker for developing atherosclerotic plaque at all phases of initiation, progression and its worst outcomes of plaque rupture [88].

The first and foremost trigger that leads to endothelial dysfunction is inflammation [85]. It plays a major role in the initiation of vascular lesions, which progresses due to the collaborative role of inflammation and endothelial dysfunction. The cellular debris generated as a consequence of inflammation-induced atherosclerosis such as vascular permeability and trapping of lipoproteins incites endothelial dysfunction [89]. Endothelial dysfunction, synchronized with inflammation, sets forth a pathological pathway comprising recruitment of monocytes from the circulating blood into the intima, transcytosis of micro-cholesterol crystals, foaming of lipid-laden cells, generation of cytokines/chemokines and synthesis of growth factors. All this contributes significantly to developing the skeleton of the plaque whereby structurally unstable plaque ruptures, which releases highly thrombogenic contents into the luminal area, triggering atherothrombotic occlusion [90]. Otherwise, if the atherosclerotic plaque is stable, then superficial plaque erosions induce apoptosis of the endothelial cells causing endothelial denudation and thrombus formation [91].

Several risk factors such as smoking, hypercholesterolemia, diabetes and hypertension participate in the generation of reactive oxygen species (ROS) within vessel walls. ROS increase oxidative stress, which in turn impairs vascular health and its function [92]. Increased vascular oxidative stress due to ROS chemically inactivates bioactive NO by producing dysfunctional superoxides and toxic peroxynitrates. This oxidative stress impairs the paracrine function of endothelial cells in regulating vasomotor function, vascular tone, platelet aggregation and proliferation of vascular smooth muscle. This way, oxidative stress-induced endothelial dysfunction promotes vasospasm, atherothrombosis and vascular inflammation [93].

Largely, all the pathological stimuli incite the endothelium to initiate vasodilation. Vasodilation is primarily carried out by the synthesis of NO, which enters endothelial intima and localizes at the layer of smooth muscle cells (SMCs) within vessel walls. In response to lesser NO bioavailability, nitrosylation of heme causes degradation of cyclic guanosine monophosphate (cGMP), which mediates the cytosolic calcium concentration and encourages the smooth muscle fibers to relax causing vasodilation. NO is produced by three isoforms of NO synthase (NOS) by using L-arginine. This reaction requires molecular oxygen as substrate, cofactors such as nicotinamide adenine dinucleotide phosphate (NADPH), flavin adenine dinucleotide (FAD), flavin mononucleotide (FMN) and tetrahydrobiopterin (BH4) [94]. During lesser production of NO, impaired vasodilation ensues due to delinked soluble guanylylcyclase/cGMP/protein kinase G cascade in SMCs. Several other processes such as uncoupling of endothelial nitric oxide synthase (eNOS), faulty phosphorylation of eNOS, inhibition of eNOS by endogenous N-methylarginine and enzymatic degradation of NO by oxidative damage and ROS generation also contribute [95,96]. eNOS expression is controlled at different levels of transcription, translation and post-translation. MicroRNAs (miRs) modulate eNOS expression post-translationally, promoting endothelial dysfunction leading to atherosclerosis. The miR-221/222 cluster influences vascular remodeling in response to vascular injury by inhibiting angiogenesis, proliferation and migration of endothelial cells [97]. Similarly, miR-195 and miR-222 promote thrombosis and are hence negatively correlated with eNOS signaling and expression [98]. Similarly, miR-92a of the miR-71 cluster inhibits angiogenesis by targeting mRNA of pro-angiogenic proteins such as integrin subunit alpha 5.

## 6. Therapies Used in Other Diseases: A Possible Avenue for ANFH Management

ANFH attracted attention when it was confirmed that people with long COVID-19 are more vulnerable to ANFH [99,100]. The primary reason identified was the use of life-saving corticosteroids for patients suffering from COVID-19 [101]. Several therapies catering to resolve pain and improve structure function have been reported [102,103,104,105,106,107]. These therapies include core decompression (CD), small-diameter drilling CD, extracorporeal shockwave therapy (EPSW), microsurgical fibula flaps (MFFs) and combination therapies such as alendronate combined with EPSW or autologous bone marrow buffy coat grafting combined with CD. All these therapies are effective in reducing lesion volume, progression of necrosis and pain and improving the endothelial functional status of the femoral head to some extent but failed to resolve necrosis. This prompted us to also look at ANFH management from the perspective of other angles, which may help provide solutions as an adjunct therapy to abovementioned invasive therapies for the proper restoration of the blood supply for cleansing, repairing and healing the necrotic head. The present study has attempted to assimilate some current reports showing therapeutic solutions for promoting angiogenesis, blocking coagulopathy and improving endothelial function in other diseases including some bone diseases, which may open new vistas for better management of patients suffering from ANFH (Table 1).

Although therapies shown in the Table 1 have been utilized for other diseases, they also have the potential to prevent necrosis of the femur head and its worst outcomes. Some pro-angiogenic therapies such as reperfusion, VEGF, stem cells, PDGF, hyperbaric oxygen and gene therapy can be adapted and applied to different phases of ANFH pathology so that it can improve and restore blood supply and facilitate tissue repair and healing. Similarly, several thrombolytic and fibrinolytic therapies used to preserve coagulation homeostasis in acute limb ischemia, intracranial artery stenosis, non-ST-segment elevated myocardial infarction and acute coronary syndromes can be utilized in attempts to resolve emboli within vessel walls feeding blood to the necrotic area of the femoral head. Likewise, several therapies have been shown to improve endothelial function in cardiovascular diseases. It is reasonable to believe that without maintaining endothelial health, restoration of the free flow of blood to the bone remains incomplete. Therefore, therapies such as antioxidant therapy, using androgen receptor agonists such as ticagrelor, infusions of NO, statins, angiotensin-II receptor blockers, CircHIPK3 therapy and L-arginine therapy may demonstrate promising effects on the recovery of endothelial function, hence promoting repair, preserving bone remodeling and potentiating healing.

## 7. Clinical Implications: A Call of a Crackling Tone of the Collapsing Bone

The present review aims to draw the attention of orthopedists, surgeons and health care providers to a deceptive but painful skeletal disorder which in its early stage is difficult to diagnose, as its pathology mimics cysts or lesions in subchondral bone, vasculitis, transient osteoporosis of the hip or osteoarthritis. Management modalities such as core depression, bone grafting, bone reshaping and bone marrow stem-cell supplementation are provided before collapse; otherwise, surgical joint replacement is the only option. Therefore, clinical implications must be harnessed so that knowledge acquired would be beneficial and directly translate to the diagnosis, prognosis and therapeutic management of ANFH.

In the early phases of ANFH, the patient feels fatigue and lethargy, which are generally considered to be the artifacts of either occupational hazards (wrong posture, prolonged load on the bone, sitting for longer durations) or affiliated complications of obesity and sedentary lifestyle. Thorough investigation of clinical chapters has suggested that intramuscular injections of vitamin B2 or implantation of cryogels containing VEGF and bone morphogenetic protein-4 (BMP-4) or administration of hyperbaric oxygen to the vessels feeding articular cartilage can trigger angiogenesis, which compensates the oxygen and nutrient demand by establishing alternative routes. Similarly, at the stage where plaque has formed and started obstructing the free flow of blood to the bone, coagulopathy is the stubborn clinical entity which needs to be resolved. Oral administration of pentosan polysulfate sodium (PPS), clopidogrel combined with aspirin or rivaroxaban or coupled therapy of anticoagulants and antiplatelets may promote thrombolysis, an invincible and imperative remedy to open obstructed areas of vasculature supplying blood to the bone. Endothelial dysfunction not only disturbs the dynamic paradigm of vasculature but also encourages the inflammatory signaling to start blocking the flow of blood.

In the clinical arena of orthopedic research, it is a compelling argument that tests and trials are essential to understanding the complex skeletal pathologies such as ANFH. With reference to this, one may have reason to believe that oral, sublingual or intravenous administration of nitric oxide; intravenous infusion of dimethyloxalyglycine; oral supplementation of Icarrin, Statins, L-arginine, or use of anticoagulants; angiotensin converting enzyme inhibitors; and angiotensin-II receptor blockers would be very beneficial for the preservation, protection and promotion of the endothelial health of the individual. Although this study puts forth a perspective that impaired angiogenesis, coagulopathy and endothelial dysfunction should be treated simultaneously because of their interactive intentions to harm the femoral head, future observational and interventional trials will clarify which therapy is most suitable at which stage.

## 8. Conclusions

The present study elaborates on the pathological events of the clinical trajectory that traverses from impaired angiogenesis, progresses via coagulopathy and worsens from endothelial dysfunction. Nonetheless, this study has a limitation, namely that only three signaling pathways were investigated, and thus it cannot be correlated with the invasive surgical techniques that correct femoral head degradation/collapse. In-depth analysis of the signaling pathways involved for the risk of ANFH suggests that a joint management regime is required to curtail the impaired angiogenesis, coagulopathy and endothelial dysfunction rather than an individual approach. This treatise exhibits some possible avenues of pharmacological interventions for alleviating risk of ANFH.

## 9. Future Directions

Owing to complex and multifactorial etiology, ANFH development has been examined from the perspective of signal transduction and signaling pathways. These pathways such as impaired angiogenesis, coagulopathy and endothelial dysfunction are under strong genetic control. Genes and genetic variants that may change the course of action from their beneficial effects to causative connotations remain to be explored thoroughly from the perspective of ANFH risk. Moreover, genes interact with locally occurring environmental factors that may enhance the severity and exacerbate the outcome of ANFH. Interestingly, response to putative drug therapies is substantially associated with the genetic endowment of the individual. Apropos of this, more than 200 FDA-approved drugs have been labeled as pharmacogenetic drugs with a note that these drugs should be prescribed according to the individual’s genetic profile (PharmGKB. Drug labels https://www.pharmgkb.org/ (accessed on 31 July 2023)). Future studies incorporating such genetic hotspots and their response to suggested therapies may expose those polygenic risk-score-based predictive markers, which can switch bone resorption and degradation to bone remodeling, leading to repair and healing of necrotic area of the femoral head. Such strategies will open new vistas of precision and personalized medicine, where every ANFH patient will be treated according to their unique genetic carriage and consequent response to that drug regimen.

## Figures and Tables

**Table 1:** Pro-angiogenesis, fibrinolytic and endothelial-function-improving therapies in several diseases.

S.No.	Therapy	Methodology	Functional Output	Authors
1.	Reperfusion therapy	Crystalloid fluid resuscitation.	Reperfusion therapy enhanced angiogenesis in a rat model of hemorrhagic shock.	Li et al. [108]
2.	BMSC-derived Li-exosome therapy	Surgical implantation of extracellular matrix-mimicking hydrogels infused engineered exosome.	BMSC-derived Li exosomes increased osteogenesis and angiogenesis in rat models of GIONFH.	Chen et al. [109]
3.	Vitamin B2 therapy	Intramuscular injection.	Vitamin B2 promoted angiogenesis in a rat model of GIONFH.	Guo et al. [110]
4.	Gene therapy	Targeted delivery of pro-angiogenic factors via plasmids.	Gene therapy induced angiogenesis in patients of RA and SLE.	Ren et al. [111]
5.	Stem-cell therapy	Targeted delivery of marrow-derived and genetically modified stem cells.	MSCs triggered angiogenesis in various pre-clinical and clinical phases of RA.	Sarsenova et al. [112]
6.	Hydrogel-based VEGF therapy	Intraperitoneal injection.	VEGF initiated angiogenesis in a rat model of MRONJ.	Sharma et al. [113]
7.	BD-2 therapy	Targeted implantation of BC-ALG-BD2 hydrogel membranes.	BD-2 prompted angiogenesis in a rat model of a calvarial defect.	Yuan et al. [114]
8.	HBOT	Oxygen administration at a pressure greater than atmospheric pressure.	HBOT developed angiogenesis in a randomized clinical trial of patients with STEMI.	Martin-Hernandez et al. [115]
9.	Growth factor therapy	Targeted implantation of cryogels infused with VEGF and BMP-4.	Coupled growth factor therapy initiated angiogenesis in a mouse model of cranial defect.	Lee et al. [116]
10.	CD34+ stem-cell-derived exosome therapy	Intravenous injection.	CD34+ stem-cell-derived exosomes triggered angiogenesis in a rat model of ONFH.	Zuo et al. [117]
11.	LLLT/PBM	Electromagnetic beam of a particular frequency and wavelength.	LLLT started angiogenesis in a randomized clinical trial of patients with STEMI.	Elbaz-Greener et al. [118]
12.	Combined growth factor therapy	Subcutaneous implantation of VEGF-BMP-2- and FGF-2-BMP-2-loaded composite scaffolds.	Combined growth factor therapy prompted angiogenesis in a rat model of a calvarial defect.	Kuttapan et al. [119]
13.	b-FGF therapy	Targeted intravenous infusion.	b-FGF initiated angiogenesis for fracture repair of the femur in a mouse model.	Zhang et al. [120]
14.	iPS-MSC-Exo therapy	Intravenous infusion.	iPS-MSC-Exo stimulated angiogenesis in a rat model of ONFH.	Liu et al. [121]
15.	Butyl 10-undecenoate therapy	Oral administration.	Butyl 10-undecenoate therapy triggered angiogenesis in a distraction osteogenesis rat model.	Ozdel et al. [122]
16.	miRNA therapy	Targeted intravenous infusions.	miR-132 induced angiogenesis in a hind–limb ischemia mouse model.	Gomes et al. [123]
17.	BMP-2 therapy	Targeted intravenous administration.	BMP-2 promoted angiogenesis in a rat model of a bone segmental defect.	Kumar et al. [124]
18.	PDGF therapy	Targeted intravenous injection.	PDGF prompted angiogenesis for fracture repair of the tibia in a rat model.	Hollinger et al. [125]
19.	Erythropoietin therapy	Targeted intravenous administration.	Erythropoietin enhanced angiogenesis for fracture repair of the right femur in a mouse model.	Holstein et al. [126]
20.	Dual growth factor therapy	Targeted delivery of BMP-2 and VEGF via retroviral vectors.	Dual growth factor therapy promoted angiogenesis in a mouse model of calvarial defects.	Peng et al. [127]
21.	VEGF therapy	Targeted intravenous infusion.	Targeted VEGF therapy induced angiogenesis in RA patients.	Ballara et al. [128]
22.	PPS therapy	Oral administration.	PPS initiated fibrinolysis in a non-randomized trial of patients with knee osteoarthritis.	Liu et al. [129]
23.	Dual-antiplatelet therapy	Oral administration of clopidogrel combined with aspirin.	Dual-antiplatelet therapy enhanced thrombolysis in a randomized trial of elderly patients with symptomatic ICAS.	Song et al. [130]
24.	Alteplase	Intravenous infusion.	Standard-dose alteplase increased fibrinolysis in acute-ischemic stroke patients in a clinical trial.	Wang et al. [131]
24.	Heparin therapy	Oral and intravenous administration.	Unfractioned heparin helped to induce thrombolysis in non-STEMI patients.	Tashani et al. [132]
25.	Urokinase	Black phosphorous nanosheet-loaded intravenous infusion.	Urokinase helped to enhance fibrinolysis in a mouse model of middle-cerebral artery occlusion.	Wang et al. [133]
26.	Tenecteplase therapy	Intravenous administration.	Tenecteplase treatment helped to promote thrombolysis in acute ischemic stroke patients.	Tsivgoulis et al., 2022, [134]
27.	Rivaroxaban therapy	Oral administration.	Rivarobaxan promoted thrombolysis in a randomized trial of chronic coronary syndrome patients.	Adik-Pathak et al. [135]
28.	Fondaparinux therapy	Subcutaneous injection.	Fondaparinux enhanced thrombolysis in acute coronary syndrome patients.	Khan et al. [136]
29.	Streptokinase therapy	Intravenous administration.	Streptokinase promoted thrombolysis in STEMI patients.	Koh et al. [137]
30.	Coupled anticoagulant therapy	Oral administration of etanercept combined with celecoxib.	Coupled anticoagulant therapy helps to induce thrombolysis in a randomized trial of patients with ankylosing spondylitis.	Tu et al. [138]
31.	Fibrinolytic factor therapy	Targeted delivery of different fibrinolytic factors.	Fibrinolytic factor therapy promoted fibrinolysis in mouse models of various bone-diseases.	Okada et al. [139]
32.	Enoxaparin therapy	Direct oral administration.	Enoxaparin reduced hypofibrinolysis in a case report of a patient with ONFH.	Haydock et al. [140]
33.	tPA therapy	Intravenous infusions, hydrogels, liposome systems.	tPA administered via liposomal drug delivery systems induced thrombolysis in ischemic stroke patients.	Fukuta et al. [141]
34.	NK1R antagonists	Oral administration.	Aprepitant stimulated fibrinolysis in patients with RA.	Liu et al. [142]
35.	MQEP therapy	Oral administration.	MQEP helped to induce fibrinolysis in patients with non-traumatic ONFH.	Li et al. [143]
36.	Desmoteplase therapy	Intravenous infusion.	Desmoteplase helped to promote thrombolysis in acute ischemic stroke patients.	Li et al. [144]
37.	Reteplase therapy	Intravenous injection.	Reteplase increased thrombolysis in acute ischemic stroke patients.	Ozluer et al. [145]
38.	Vitamin E therapy	Oral and intravenous delivery.	Vitamin E helped to start fibrinolysis in osteoarthritis patients.	Li et al. [146]
39.	NO donors	Oral, sublingual and intravenous administration.	NO donors helped to increase endothelial function in ischemia-reperfusion injury in multiple randomized clinical trials.	Dou et al. [147]
40.	Dimethyloxalylglycine	Intravenous infusion.	Dimethyloxalylglycine enhanced endothelial function in a rat model of ONFH.	Shao et al. [148]
41.	CircHIPK3 therapy	Targeted intravenous injection.	CircHIPK3 improved endothelial function in patients with ONFH.	Peng et al. [149]
42.	*Chromolaena odarata* therapy	Oral administration of aqueous extract.	*Chromolaena odarata* extract helped to induce endothelial function in a rat model of ONFH.	Nguenum et al. [150]
43.	Icariin therapy	Oral administration.	Icariin helped to increase endothelial function in osteonecrosis and osteoporosis patients.	Zhang et al. [151]
44.	Statins	Oral administration.	Statins improved endothelial function in ONFH patients and in vivo studies.	Yu et al. [152]
45.	Tissue regeneration therapy	Targeted delivery of BMP-2 via PEM-coated scaffolds.	Tissue regeneration therapy enhanced endothelial function in a rat model of calvarial defects.	Martin et al. [153]
46.	PTEN inhibitors	Intravenous infusion.	VO-OHpic reduced endothelial dysfunction in an in vivo study of an ONFH animal model.	Yao et al. [154]
47.	L-Arg therapy	Oral and intravenous administration.	L-Arg promoted endothelial function in ischemic diseases in various clinical trials.	Gamberdella et al. [155]
48.	ROS inhibitors	Oral and intravenous infusion.	ROS inhibitors attenuated endothelial dysfunction in multiple bone disorders.	Agidigbi et al. [156]
49.	Se@SiO_2_ nanocomposites therapy	Intraperitoneal injection.	Se@SiO_2_ nanocomposites lessened endothelial dysfunction in rat models of ONFH.	Deng et al. [157]
50.	Antioxidant therapy	Oral and intravenous administration.	Antioxidant therapy improved endothelial function in hip fracture patients.	Sprague et al. [158]
51.	ARBs	Oral and intravenous infusion.	ARBs helped to reduce endothelial dysfunction in various clinical trials of different cardiovascular diseases.	Radenkovic et al. [159]
52.	ACE inhibitors	Oral administration.	ACE inhibitors increased endothelial function in a randomized controlled trial of T2DM patients with myocardial infarction.	Sun et al. [160]
53.	ET-1 therapy	Intravenous injection.	ET-1 improved endothelial function in in vivo studies using recombinant endothelial progenitor cells and osteoblasts.	Wang et al. [161]
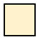 **Indicates pro-angiogenic therapies** 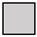 **Indicates fibrinolytic therapies** 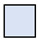 **Therapies for improving endothelial function**.				

**BMSC:** bone-marrow-derived stem cell, **GIONFH**: glucocorticoid-induced osteonecrosis of femoral head, **OTF-PNS/nHAC/Mg/PLLA**: osteopractic total flavone—panax notoginseng saponin/nano-hydroxyapatite collagen/magnesium/poly (L-lactic acid), **Cu-Li-nHA**: copper–lithium-doped nanohydroxyapatite, **ONFH**: osteonecrosis of femoral head, **RA**: rheumatoid arthritis, SLE: systemic lupus erythematous, MSCs: mesenchymal stem cells, VEGF: vascular endothelial growth factor, **MRONJ**: medication-related osteonecrosis of jaw, **BD-2**: beta-defensin-2, **HBOT**: hyperbaric oxygen therapy, **STEMI**: ST-segment elevation myocardial infarction, **BMP-4**: bone morphogenetic protein-4, **CD34+**: cluster of differentiation 34+, **LLLT**: low-level laser therapy, **PBM**: photobiomodulation, **FGF-2**: fibroblast growth factor-2, **BMP-2**: bone morphogenetic protein-2, **b-FGF**: basic fibroblast growth factor, **iPS-MSC-Exos**: induced pluripotent stem-cell-derived mesenchymal cells, **mi-RNA**: micro-RNA, **PDGF**: platelet-derived growth factor, **PPS**: pentosanpolysulfate sodium, **ICAS**: intracranial artery stenosis, **LMWHs**: low-molecular-weight heparins, **tPA**: tissue–plasminogen activator, **NK1R**: neurokinin-1 receptor, **MEQP**: modified Qing’e pill, **NO**: nitric oxide, **CircHIPK3**: circular RNA homeodomain-interacting protein kinase 3, **HF-ESWT**: high-energy focused extracorporeal shock wave therapy, **PEM**: polyelectrolyte multilayer **PTEN**: phosphate and tensin homolog deleted on chromosome 10, **VO-OHpic**: 3-hydroxypicolinate vanadium, **Arg**: arginine, **ROS**: reactive oxygen species, **Se@SiO**: Se.particle@porous.silica, **ARBs**: angiotensin-II receptor blockers, **ACE**: angiotensin-converting enzyme, **T2DM**: type 2 diabetes mellitus, **ET-1**: endothelin-1.

## Data Availability

Data regarding this paper is available with the corresponding author.
